# Hypophosphatemia in Patients Receiving Intravenous Iron Supplementation for Iron-Deficiency Anemia: A Narrative Review

**DOI:** 10.3390/jcm15124748

**Published:** 2026-06-18

**Authors:** Giovanni Inghilleri, Massimo Franchini

**Affiliations:** 1 Department of Immunohematology and Transfusion Medicine, Fatebenefratelli Sacco, 20157 Milan, Italy; 2 Department of Hematology and Transfusion Medicine, Carlo Poma Hospital, 46100 Mantua, Italy; massimo.franchini@asst-mantova.it

**Keywords:** ferric carboxymaltose, ferric derisomaltose, hypophosphatemia, intravenous iron, iron-deficiency anemia

## Abstract

Intravenous (IV) iron is used to replenish iron stores in patients with iron-deficiency anemia (IDA) who do not benefit from oral iron supplementation. Hypophosphatemia is an increasingly recognized adverse event associated with certain IV iron formulations. Mild/moderate hypophosphatemia may be asymptomatic or present with symptoms similar to those seen in patients with IDA, including fatigue, malaise, and muscle weakness. Persistent hypophosphatemia can cause osteomalacia due to reduced bone mineralization, leading to bone pain and pseudofractures. Ferric carboxymaltose (FCM) can impact phosphate homeostasis through an increase in fibroblast growth factor 23, leading to increased urinary phosphate excretion and hypophosphatemia. In clinical trials, rates of hypophosphatemia were significantly higher in patients receiving FCM compared with other IV iron formulations, such as ferric derisomaltose and ferumoxytol. Treatment guidelines recommend monitoring serum phosphate levels in patients receiving FCM who are at risk for low phosphate or who require repeat infusions, and alternative iron formulations should be considered in at-risk patients. This narrative review summarizes current evidence regarding IV iron-induced hypophosphatemia in individuals with IDA and examines the underlying pathophysiology and clinical evidence for IV iron-induced hypophosphatemia, particularly with FCM, the populations most at risk, and the clinical consequences of persistent hypophosphatemia.

## 1. Introduction

There are an estimated 1.8 to 1.9 billion prevalent cases of anemia worldwide, and iron deficiency, whether due to excessive bleeding, inadequate dietary iron intake, or inflammation and hepcidin blockage of iron body stores, is the primary etiology [1,2,3]. In particular, the role of hemojevulin within the hepcidin/ferroportin axis has recently been revealed as a key regulator controlling iron sequestration in anemia of inflammation [4,5]. The principal causes of blood loss contributing to iron-deficiency anemia (IDA) are menstrual bleeding in premenopausal women and gastrointestinal blood loss in men and postmenopausal women [3]. IDA is one of the leading causes of years lived with disability and, as such, represents a substantial global health care burden [6,7]. Clinically, IDA, if untreated, is a chronic condition that can be symptomatic or asymptomatic. Nonspecific symptoms may include weakness, fatigue, lethargy, impaired concentration, and headache [3,8]. Iron deficiency can also result in impaired health-related quality of life, even in the absence of anemia [8]. In the United States, approximately 39% of females aged 12 to 21 years have iron deficiency and 6.3% have IDA [9]. In Italy, the incidence of IDA increased by >50% between 2002 and 2013 and was almost 4 times higher in females compared with males [10]. The prevalence of IDA in Italy in 2013 was 1.92% (0.77% in males and 2.91% in females) [10]. Globally, prevalent cases of iron deficiency are projected to reach 1400 million by 2050 [6].

Oral iron supplementation is recommended as the first-line treatment for patients with IDA, and intravenous (IV) iron is recommended for those who cannot tolerate oral iron, whose iron levels do not recover with oral supplementation, or in those who are unlikely to absorb oral iron [11,12]. IV iron can also be used when urgent correction of IDA is required [11,12]. The overall aim of iron supplementation is to correct anemia, replenish iron stores, improve symptoms when present, and restore quality of life [13]. Several IV iron formulations are now available that differ in terms of dose and frequency but are considered broadly equivalent in terms of hematologic response per gram of iron [11,12]. Hemoglobin (Hb) levels typically can be expected to increase by 2 g/dL within 3 to 4 weeks following IV iron administration [14]. One feature that is increasingly recognized to differ across different IV iron formulations, however, is the incidence of treatment-related hypophosphatemia [15,16]. Low serum phosphate levels following IV iron infusion are frequently asymptomatic, but severe cases associated with significant clinical sequelae have also been reported, particularly with persistent hypophosphatemia [15].

Hypophosphatemia (serum phosphate < 2.5 mg/dL or <0.80 mmol/L) can manifest in many different clinical scenarios [17,18,19,20]. Broadly, it can be categorized under 3 different etiologies as (1) inadequate intake or absorption, (2) redistribution to the intracellular space or bone, and (3) renal phosphate loss [18]. Inadequate intake can arise due to malabsorption or low dietary phosphate availability, and redistribution to the intracellular compartment occurs in patients with diabetic ketoacidosis [18]. Clinically, hypophosphatemia is often seen in critically ill patients and is a common manifestation among patients admitted to intensive care units (ICUs) [21]. In these patients, underlying causes of hypophosphatemia can include acute respiratory alkalosis, alcoholism and vomiting, or gastric losses [21]. Among critically ill patients with major trauma, hypophosphatemia is associated with a significant increase in duration of stay in the ICU [22]. Hypophosphatemia or elevated urinary phosphate loss may also occur in patients following theophylline or acetaminophen overdose, those receiving parenteral feeds including amino acids, those with extensive third-degree burns, or those with diabetes who develop glycosuria, ketonuria, or polyuria [23]. Individuals with chronic alcohol use disorder may also present with hypophosphatemia due to poor nutritional status or diarrhea and vomiting. Chronic alcohol use and poor diet may also lead to repeated ketoacidosis, which may precipitate phosphate loss similar to that seen in diabetic ketoacidosis and ultimately lead to osteomalacia [23,24].

The aim of this review is to appraise current evidence regarding IV iron-induced hypophosphatemia in individuals with IDA. We will examine the underlying pathophysiology and clinical evidence for IV iron-induced hypophosphatemia in patients with IDA, the populations most at risk, and the clinical consequences of persistent hypophosphatemia. We will also review the occurrence of hypophosphatemia with structurally diverse IV iron formulations and describe the clinical recommendations that should be followed to prevent development of hypophosphatemia.

## 2. Methods

This was a narrative review, and systematic search methodologies were not employed. PubMed was searched for relevant articles using the following keywords alone and in combination: “hypophosphatemia”, “intravenous iron-supplementation” “ferric carboxymaltose”, “ferric derisomaltose”, “clinical trial”, “randomized clinical trial”, “phosphate”, “fibroblast growth factor”, and “guidelines”. Further articles were selected for inclusion based on a manual review of these search results and their reference lists. Pre-defined search criteria were not employed, and articles were selected for inclusion based on the clinical experience of the authors. All reasonable efforts were made to ensure that articles selected for inclusion were representative of the wider clinical literature and were consistent with the experience of the authors in this therapeutic area; however, the lack of a systematic search strategy may result in some selection bias.

## 3. IV Iron Supplements

Delivery of IV iron supplementation has improved with the availability of third-generation compounds, including ferumoxytol, iron isomaltoside (ferric derisomaltose [FDI]), and ferric carboxymaltose (FCM). The use of early IV iron formulations was associated with rapid labile iron release, leading to poor tolerability, including serious toxic reactions [15]. Subsequent formulations were developed to incorporate a carbohydrate coating surrounding the iron core to slow the release of iron. However, reports of anaphylactic reactions limited the use of these second-generation formulations and precipitated further molecular refinements to improve tolerability. The third-generation agents incorporate carbohydrate-stabilized polynuclear Fe (III)-oxyhydroxide/oxide nanoparticles formulated within colloidal solutions [13,15]. Tighter binding of elemental iron within the iron-carbohydrate complex reduces both toxicity from labile iron release and immunogenic activity, leading to a lower risk of serious hypersensitivity reactions [15].

Each of the third-generation IV iron formulations has differing chemical and physical properties, including the carbohydrate moiety, iron oxyhydroxide structure, molecule size, and surface charge [15]. FDI includes a derisomaltose carbohydrate, which consists of linear, unbranched hydrogenated isomalto-oligosaccharides with an average molecular weight of 1 kDa (corresponding to 5–6 glucose units) [15]. Ferumoxytol contains a polyglucose sorbitol carboxymethyl ether produced from dextran, which is a predominantly linear glucose polysaccharide with a low degree of branching and a molecular weight of approximately 10 kDa [15]. The carboxymaltose moiety in FCM is derived from a commercially available maltodextrin and consists of 520 glucose units with a predominantly linear backbone and branches that are mainly α-(1,6)-linked [15]. The greater stability of the carbohydrate shell in these third-generation formulations permits delivery of a full dose in 1 or 2 infusions while minimizing the release of free iron and reducing rates of anaphylaxis compared with older formulations [25].

The different third-generation formulations are all generally comparable in terms of safety, including infusion reactions [11,12,26]. The most common infusion reaction categorized as an adverse event with IV iron is the Fishbane reaction, which is believed to be triggered by labile iron [27]. This is usually a self-limited response to IV iron that can be managed by pausing the infusion and restarting at a slower rate. Symptoms include flushing, myalgia/arthralgia, and back pain/chest pressure [27]. Complement activation-related pseudoallergy (CARPA) infusion reactions, which cause similar symptoms as Fishbane reactions, are mediated through the activation of the complement by labile iron, leading to mast cell degranulation. In contrast, allergic immunoglobulin E (IgE)-mediated hypersensitivity reactions require prior sensitization to iron and occur when iron re-exposure cross-links IgE, causing mast cell degranulation. IgE-mediated hypersensitivity can lead to severe symptoms of airway compromise, mucosal swelling, circulatory manifestations, and gastrointestinal symptoms and, ultimately, life-threatening anaphylaxis [17]. However, most infusion reactions to IV iron, including Fishbane reactions and CARPA infusion reactions, have not been reported to be a consequence of IgE sensitization [28]. Extravasation, or the release of IV fluid from the vein into the surrounding tissue at the administration site, is an uncommon infusion-related complication that has been reported with IV iron [29,30,31]. Hypophosphatemia represents the most widely reported tolerability concern with IV iron supplementation that differs between IV iron formulations [15,16].

## 4. Role of Phosphate in the Human Body and Phosphate Homeostasis

Phosphorus is highly reactive and does not exist in its elemental form in the human body [32]. Rather, it exists as organic phosphate as a component of phospholipids, nucleic acids, and phosphoproteins and as inorganic phosphate, which acts as a substrate for energy generation, such as in the synthesis of adenosine triphosphate [33,34]. Phosphate is absorbed by active and passive transport from the gastrointestinal tract and is excreted via the kidneys, where 80% to 90% of the inorganic phosphate filtered by the glomeruli is reabsorbed via sodium-phosphate cotransporters in the proximal tubules and the remainder is excreted in the urine [33,34,35]. Of the 600 to 800 g of phosphate found in the human body, 80% to 85% is found in bone and teeth complexed with calcium as hydroxyapatite, 10% to 15% in soft tissue, and ~1% in the extracellular fluid [33,34]. Organic phosphate is used in enzyme processes including oxidative phosphorylation, glycolysis, and ammoniagenesis, and phosphate also influences the oxygen-carrying capacity of Hb through regulation of 2,3-diphosphoglycerate synthesis [33]. In the extracellular fluid, inorganic phosphate (H_2_PO_4_^−^ or HPO_4_^2−^) is used as a buffer and as a regulator of mineralization [33].

**Figure 1:**
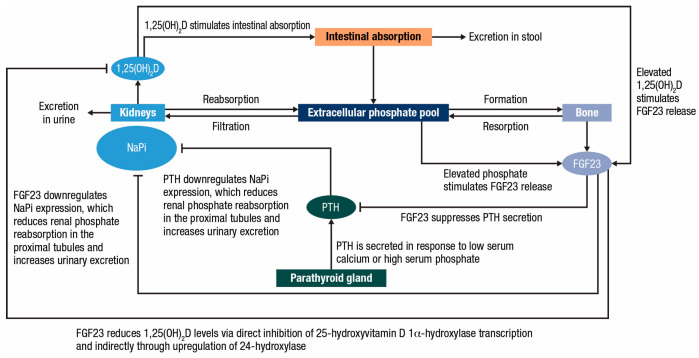
Overview of phosphate homeostasis. 1,25(OH)_2_D, 1,25-dihydroxyvitamin D; FGF23, fibroblast growth factor 23; NaPi, sodium-phosphate cotransporter; PTH, parathyroid hormone.

Phosphate homeostasis is controlled by fibroblast growth factor 23 (FGF23), parathyroid hormone, and 1,25-dihydroxyvitamin D, the biologically active form of vitamin D (Figure 1). FGF23 is produced primarily in mineralized tissues, such as osteoblasts, and is synthesized in response to dietary intake of phosphorus or elevated serum levels of phosphate or 1,25-dihydroxyvitamin D. FGF23 regulates serum phosphate concentrations by reducing renal phosphate reabsorption in the proximal tubules through a decrease in the expression of the sodium-phosphate cotransporters [36,37]. FGF23 also regulates vitamin D homeostasis directly by inhibiting transcription of 25-hydroxyvitamin D 1α-hydroxylase and indirectly through upregulation of 24-hydroxylase, both leading to reduced concentrations of 1,25-dihydroxyvitamin D [36,38]. Reduced 1,25-dihydroxyvitamin D levels cause mild hypocalcemia due to reduced calcium absorption from the gut and result in an increase in circulating levels of parathyroid hormone. The phosphaturic effects of parathyroid hormone can then further prolong hypophosphatemia beyond the initial period of elevated FGF23 by also reducing phosphate reabsorption in the proximal tubules through downregulation of sodium-phosphate cotransporters [39].

## 5. Mechanism of FCM-Induced Hypophosphatemia

In patients with IDA, supplementation with certain IV iron formulations, particularly FCM, is associated with an increase in circulating FGF23 levels, which leads to hypophosphatemia [40,41,42,43,44]. Intact FGF23 levels increase by 3- to 6-fold within 24 h following infusion of FCM, but similar increases are not seen following infusion of FDI or ferumoxytol, suggesting that this effect is specific to the unique chemical properties of FCM [45]. Increased serum FGF23 levels following FCM administration result in increased renal phosphate excretion both via a direct effect at the proximal tubules and indirectly by inhibiting the activation of 1,25-dihydroxyvitamin D; this leads to increased parathyroid hormone and secondary hyperparathyroidism, which then further exacerbates phosphate excretion, even when levels of intact FGF23 have normalized [16,45,46]. The exact mechanism by which FCM causes an increase in serum FGF23 is unknown, but it has been proposed that FCM acts by preventing the cleavage of intact FGF23 into its constituent C- and N-terminal fragments [16,47]. This hypothesis is supported by the concurrent decrease in C-terminal FGF23 that accompanies increased levels of intact FGF23 following FCM administration [46]. FCM may mimic the inherited condition of autosomal dominant hypophosphatemic rickets, which is caused by missense mutations in the *FGF23* gene, resulting in mutant FGF23 that cannot be cleaved, excess FGF23, hypophosphatemia, and low 1,25-dihydroxyvitamin D levels [48].

The effect of FCM on FGF23 cleavage may be more pronounced in individuals with iron deficiency [46]. Iron deficiency may itself promote transcription of *FGF23* in the osteocyte, leading to increased production of FGF23, which is paralleled by increased degradation of FGF23. The resulting levels of intact FGF23 remain within normal ranges (and hence normal phosphate balance is maintained), but there is an increase in levels of the degradation product C-terminal FGF23 [46,47]. Inhibition of FGF23 cleavage by FCM under conditions where there is an underlying increase in *FGF23* transcription due to IDA may then drive the dramatic increase in intact FGF23 seen following administration of FCM [46].

The full cascade of events that lead to the long-term biochemical changes induced by FCM is referred to as 6H syndrome (high FGF23, hypophosphatemia, hyperphosphaturia, hypovitaminosis D, hypocalcemia, and secondary hyperparathyroidism). This cascade is thought to be unique to FCM, and while ferumoxytol and FDI may also cause hypophosphatemia at a lower frequency and severity compared with FCM, these compounds do not induce 6H syndrome [15].

## 6. Hypophosphatemia in Patients with IDA Following IV Iron Supplementation: Clinical Experience

The PHOSPHARE IDA04 and IDA05 clinical studies were 2 identical, open-label, randomized trials conducted across 30 centers in the United States [44]. Participants with IDA, defined as Hb ≤ 11 g/dL and serum ferritin ≤ 100 ng/mL, received FDI (1000 mg single dose infused over 20 min on day 0) or FCM (750 mg on day 0 and 750 mg on day 7) per their respective approved labeling (Table 1) [44]. The primary endpoint was the incidence of hypophosphatemia, defined as a serum phosphate level < 2.0 mg/dL at any time from baseline to day 35. All endpoints in these studies were biochemical assessments of serum variables; assessment of longer-term clinical outcomes was precluded by the short follow-up duration of these studies (35 days). The protocol for each study prespecified a combined analysis of the data obtained from the 2 clinical trials [44]. Participants were primarily female with IDA due to gynecological bleeding.

In both trials, the incidence of hypophosphatemia through day 35 was significantly lower in participants receiving FDI than in those receiving FCM, with incidence rates of 7.9% versus 75.0% in PHOSPHARE IDA04 (adjusted rate difference: −67.0% [95% confidence interval (CI), −77.4%, −51.5%]; *p* < 0.001) and 8.1% vs. 73.7% in PHOSPHARE IDA05 (adjusted rate difference: −65.8% [95% CI, −76.6%, −49.8%]; *p* < 0.001), respectively [44]. Severe hypophosphatemia (serum phosphate ≤ 1.0 mg/dL) was observed in 11.3% and 0% of participants (*p* < 0.001) receiving FCM and FDI, respectively [44,50]. Moreover, only FCM-treated participants (40%) developed persistent hypophosphatemia (<2.0 mg/dL at day 35 after developing incident hypophosphatemia between days 1 and 14) [50].

Following the first dose of FCM, intact FGF23 levels increased through day 8 (day after the second dose of FCM) and thereafter decreased gradually through day 35. Intact FGF23 levels were higher in the FCM group than in the FDI group at all postbaseline assessments. Concentrations of C-terminal FGF23 decreased within 24 h of dosing in both treatment groups but then increased between days 8 and 21 in participants treated with FCM, coinciding with the peak in intact FGF23 at day 8 in FCM-treated participants. Throughout the study period, urinary phosphate excretion was significantly higher in participants receiving FCM compared with FDI [44]. Levels of 1,25-dihydroxyvitamin D and ionized calcium were also significantly lower following dosing with FCM compared with FDI, leading to secondary hyperparathyroidism, which maintained renal phosphate wasting and hypophosphatemia even after intact FGF23 levels began to reduce [44]. Hb levels, Hb per gram of iron infused, and ferritin and transferrin saturation were each increased in both treatment arms, indicative of their comparable hematologic efficacy. At day 35, increases in mean hemoglobin per gram of iron in dose were similar with FDI and FCM (2.2 g/dL [SD 1.4] vs. 2.0 g/dL [SD 0.9]). Adverse events were more common with FCM than with FDI (PHOSPHARE IDA04: 45% vs. 11%; PHOSPHARE IDA05: 49% vs. 23%), largely attributable to events of hypophosphatemia and decreased blood phosphate. Adverse events of nausea were reported in 7% of participants receiving FCM and <1% of those receiving FDI, and serious or severe hypersensitivity reactions (dyspnea and swelling) occurred in 2 (2%) participants receiving FCM and 1 (<1%) participant receiving FDI (unilateral swollen eyelid) [44].

IDA is also a common manifestation of inflammatory bowel disease, with high-dose IV iron recommended in the European Crohn’s and Colitis Organisation guidelines on IDA management as first-line treatment for patients with clinically active inflammatory bowel disease, for patients with previous intolerance to oral iron, and in patients who need erythropoiesis-stimulating agents [51]. In the PHOSPHARE-IBD study, participants with IDA due to inflammatory bowel disease (Hb < 13 g/dL and serum ferritin ≤ 100 ng/mL) and a history of intolerance or unresponsiveness to oral formulations received either FDI or FCM (Table 1) [49]. In both treatment arms, the total iron requirement at baseline was calculated based on body weight and Hb concentration [49,51]. Participants with baseline Hb < 10 g/dL received a total iron dose of 1500 mg if their body weight was <70 kg or 2000 mg if their body weight was ≥70 kg; patients with baseline Hb ≥ 10 g/dL received a total iron dose of 1500 mg. Both FCM and FDI were administered as split doses with a maximum individual dose of 1000 mg in accordance with the approved FCM dosing schedule (the maximum single FCM dose is 1000 mg). Participants received a single 20-min IV infusion of FDI or FCM (1000 mg) at baseline and a second iron dose of either 500 mg or 1000 mg (according to total calculated iron dose) at day 35 [49].

Hypophosphatemia (the primary outcome, defined as serum phosphate < 2.0 mg/dL from day 0 to day 35) was significantly higher in participants receiving FCM compared with FDI (51.0% vs. 8.3%; adjusted risk difference: −42.8% [95% CI, −57.1%, −24.6%]; *p* < 0.0001) [49]. The incidence of hypophosphatemia with FCM was highest on day 14 after the initial dose, and the risk of hypophosphatemia was similar regardless of diagnosis of Crohn’s disease or ulcerative colitis. Following administration of the second iron dose on day 35, incidence rates of hypophosphatemia at any time from baseline to day 70 were also significantly higher with FCM compared with FDI (59.2% vs. 12.5%; adjusted risk difference: −46.6% [95% CI, −60.9%, −28.1%]; *p* < 0.0001). Intact FGF23 serum concentrations and urinary phosphate excretion were significantly higher and 1,25-dihydroxyvitamin D concentrations were significantly lower following both infusions with FCM compared with FDI, and the effects on ferritin, transferrin saturation, and Hb were similar with both treatments [49]. Both iron formulations caused robust and comparable increases in Hb levels at study day 70 (FDI, 24.9 g/L [95% CI 21.1, 28.8; FCM, 25.2 g/L [95% CI 21.3, 29.1). As in the PHOSPHARE IDA04 and IDA05 studies, assessment of any longer-term clinical consequences associated with the biochemical changes in serum phosphate levels was precluded by the relatively short study duration (70 days).

Both agents also improved fatigue symptoms as measured using the Functional Assessment of Chronic Illness Therapy—Fatigue Scale, but the magnitude of improvements was greater and faster with FDI than with FCM. There was also an inverse association between improvement in fatigue and the magnitude of decrease in phosphate concentration, with slower improvement in fatigue observed among individuals with more severe hypophosphatemia [49]. Changes in health-related quality of life were similar in FCM- and FDI-treated individuals; however, improvements in the vitality subscale were significantly greater with FDI than with FCM [52]. In a pooled analysis of the FCM and FDI treatment arms, participants with the greatest decrease in phosphate level also had the lowest increase in vitality score; conversely, those with the lowest decrease in phosphate level exhibited the greatest improvement in vitality score [52].

Several other clinical studies have also reported significantly higher rates of hypophosphatemia in participants receiving FCM compared with FDI or other iron formulations [53,54,55]. The single-center HOMe aFers trial assessed the incidence of IV iron-induced hypophosphatemia in females with IDA (Hb < 12 g/dL and serum ferritin ≤ 100 ng/mL or serum ferritin ≤ 300 ng/mL with transferrin saturation ≤ 30%) due to uterine bleeding for whom oral iron supplementation was either ineffective or not tolerated [53]. Hypophosphatemia occurred in 75% of participants receiving FCM compared with 8% of those who received FDI and was accompanied by a significant increase from baseline in intact FGF23 among FCM- but not FDI-treated participants [53]. In the randomized, double-blind FIRM trial in adults with IDA (Hb < 12 g/dL [women], <14 g/dL [men], transferrin saturation ≤ 20% or ferritin ≤ 100 ng/mL, and prior intolerance or inadequate response to oral iron), the incidence of hypophosphatemia (<2 mg/dL) was higher with FCM compared with ferumoxytol (50.8% vs. 0.9%). Additionally, intact FGF23 levels were increased with FCM, but not with ferumoxytol [56]. In a study of participants with IDA of any etiology, rates of hypophosphatemia (assessed as a secondary endpoint) at 2 weeks after treatment were 38.7% with FCM compared with 0.4% in participants receiving ferumoxytol, with lower serum phosphate levels and higher fractional phosphate excretion in the FCM group. Iron, transferrin saturation, and Hb values were increased similarly in both treatment groups, and total iron-binding capacity decreased similarly in both groups [54].

In the placebo-controlled PREVENTT trial, administration of FCM (as a single 1000 mg dose) prior to elective major abdominal surgery had no impact on the composite primary endpoint of blood transfusion or death compared with placebo [57]. However, a subsequent exploratory analysis revealed that FCM-treated participants had a 9-fold increased risk of preoperative hypophosphatemia that was associated with significantly increased hospital stay duration, decreased days alive and out of hospital, and increased postoperative adverse events [58]. Finally, real-world studies report incidence rates of moderate/severe hypophosphatemia following FCM administration of 34% to 51% and rates of severe or profound hypophosphatemia of 7% to 13% [59,60,61]. Systematic literature reviews suggest that up to 92% of patients may experience hypophosphatemia following FCM administration and that FCM-induced hypophosphatemia can be persistent and last anywhere from weeks to months [16,62,63]. It is important to note that in general, these studies report biochemical changes in serum phosphate levels and do not provide insight into any longer-term clinical consequences of hypophosphatemia induced by FCM.

## 7. Symptomatic Hypophosphatemia: Clinical Presentation

**Figure 2:**
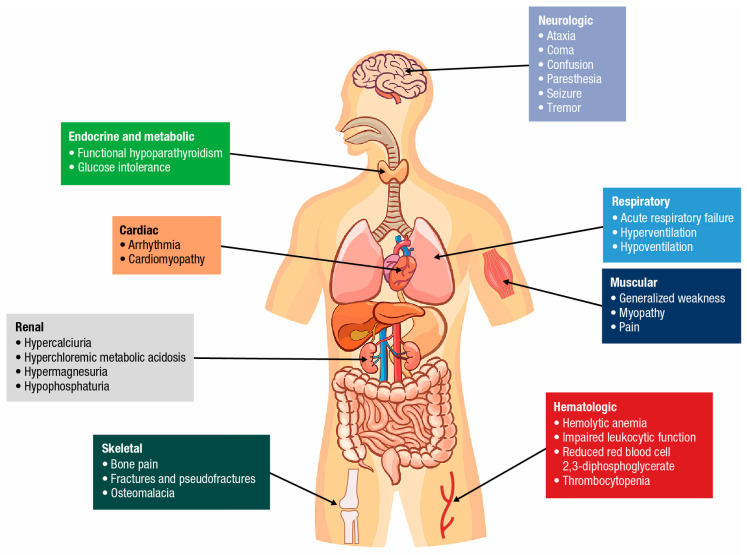
Manifestations of severe hypophosphatemia.

There is a lack of consensus on the classification of severity of hypophosphatemia, but reports have defined it as the following: mild (phosphate level: 0.80 to 0.65 mmol/L or 2.5 to 2.0 mg/dL), moderate (<0.65 to 0.35 mmol/L or <2.0 to 1.0 mg/dL), and severe (<0.35 mmol/L or <1.0 mg/dL) [17,18,19,20]. Severe or persistent hypophosphatemia can have several important physiological consequences (Figure 2). Mild/moderate hypophosphatemia may be asymptomatic and self-limited and is often detected as an incidental finding [17,40]. Differential diagnosis of acute hypophosphatemia can be complicated due to the overlapping symptoms with IDA, which include worsening fatigue/malaise, muscle weakness, myalgia, and, occasionally, gait disturbances [17,63]. Neurologic symptoms, such as paresthesias, dysarthria, altered mental status, and neuropathy may manifest in patients with severe hypophosphatemia but are rarely apparent in patients at diagnosis. Acute severe hypophosphatemia can cause impaired cardiac and respiratory function and may be life-threatening [40].

Osteomalacia is the main long-term complication of persistent severe hypophosphatemia, which leads to reduced mineralization and is characterized by muscle weakness, bone pain, pseudofractures, and elevated alkaline phosphatase activity. Most reports of osteomalacia induced by iron occur after repeated FCM use involving > 5 infusions [63]. In fact, the median number of infusions in a case series of patients with osteomalacia primarily induced by FCM was 17 infusions (range: 2–60) [63]; however, significant alterations in markers of bone mineralization and mineral metabolism have been reported following as few as 2 infusions of FCM [64]. In most cases, osteomalacia is accompanied by bone pain and is associated with fractures or pseudofractures, frequently of the chest, lower limbs, and pelvis [63]. Fractional urinary phosphate excretion and alkaline phosphatase levels are typically elevated, and N-terminal procollagen-1 peptide is decreased [50,63]. The risk of osteomalacia and fracture was substantially higher with FCM compared with FDI as demonstrated in an analysis of electronic health records, which reported a hazard ratio for fracture risk of 4.54 for FCM versus FDI [65]. In this analysis, FCM was also associated with an increased incidence and severity of hypophosphatemia and kidney stones compared with FDI; the rates for fractures were similar before and after FDI [65]. In a separate retrospective analysis of electronic health records, FCM treatment was associated with a significantly higher incidence of bone events (hazard ratio: 3.08), defined as first bone fracture or radiological sign of osteomalacia over 7 years of follow-up [66]. These findings were substantiated in an analysis of patients in the TriNetX database, demonstrating an increased risk of incident fractures with FCM vs. FDI (hazard ratio: 2.03) through 6 months of follow-up [66]. In a mouse model of IDA, FCM was found to preferentially localize to bone, resulting in reduced collagen production and ossification, which may contribute to the increased fracture risk [66].

## 8. Expert Recommendations for Managing Hypophosphatemia

There is no standard-of-care treatment for hypophosphatemia induced by FCM, and both oral and IV phosphate supplementation have been shown to be suboptimal in some patients, such as those with malabsorptive disorders or renal wasting [67,68]. Expert consensus guidelines recommend against the use of prophylactic oral phosphorus supplementation following IV iron administration due to the increase in urinary phosphate excretion that follows oral phosphate loading [69]. Indeed, phosphate replacement may increase serum parathyroid hormone levels and perpetuate existing phosphaturia. However, these guidelines are not universally adopted and some clinicians support the use of phosphate supplementation in patients with moderate/severe FCM-induced hypophosphatemia, while noting the associated risks of adverse tolerability including hypocalcemia, arrythmias and acute nephropathy, and the potential for increased parathyroid hormone release [20,70]. Mild/moderate hypophosphatemia can be asymptomatic and self-limiting, and treatment with vitamin D supplementation can be considered to mitigate secondary hyperparathyroidism [17]. Vitamin D supplementation prior to FCM administration, however, does not reduce the risk of hypophosphatemia. Additionally, coadministration of oral phosphate with FCM does not prevent FCM-induced hypophosphatemia [71].

**Figure 3:**
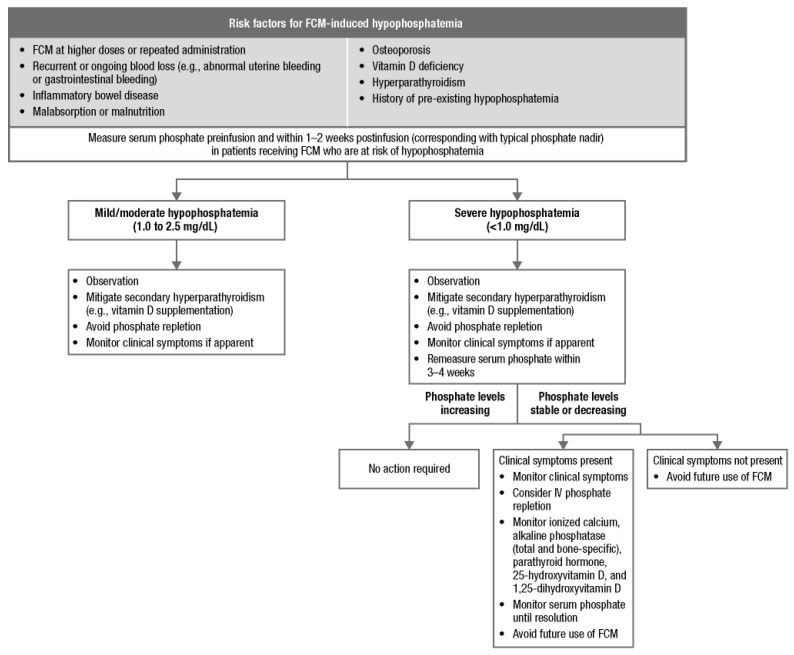
Patients at risk for FCM-induced hypophosphatemia, phosphate monitoring, and hypophosphatemia management recommendations [45,63,69,76]. FCM, ferric carboxymaltose.

There is strong evidence that the risk of hypophosphatemia associated with FCM is an important consideration when treating patients with IDA, as reflected in FCM-containing IV iron product labels [72,73]. Avoidance of FCM is the single most important intervention in individuals who have experienced FCM-induced hypophosphatemia, and alternative iron formulations to FCM should be used in patients considered at high risk (Figure 3), including those with recurrent blood loss or malabsorption, hyperparathyroidism, vitamin D deficiency, malnutrition, inflammatory bowel disease, osteoporosis, and a history of preexisting hypophosphatemia [17,69,74,75]. In a clinical trial of participants with IDA receiving FCM or ferumoxytol, the strongest risk factor for developing hypophosphatemia was treatment with FCM. Other risk factors independently associated with elevated risk for hypophosphatemia included higher estimated glomerular filtration rate, lower body weight and serum phosphate, and IDA due to abnormal uterine bleeding [56].

Consistent with the product labels, serum phosphate levels should be monitored regularly in at-risk patients receiving FCM, but universal monitoring is not required in patients receiving FDI [72,73,77,78], although it may be considered in patients where clinical symptoms exist and in those requiring repeated iron infusions at high dosage and short intervals [17,79]. Serum phosphate levels should be routinely monitored in patients receiving FCM who are at risk for low phosphate and who require repeat infusions [72,73]. Imaging should be considered for patients who report bone pain suggestive of osteomalacia [17,63]. The risks of hypophosphatemia and clinical guidelines for using FCM should be recognized for all FCM-containing IV iron supplements. High-risk patients receiving FCM as a single dose should have their phosphate level measured preinfusion and at 2 weeks postinfusion, coinciding with the typical phosphate level nadir following FCM treatment. If 2 infusions of FCM are required to achieve the target dose, the second dose should be withheld if there is evidence of hypophosphatemia after the first dose [76].

Dosing recommendations for FDI and FCM in Europe and the United States are summarized in Table 2. In both regions, FCM can be administered as a single infusion up to a maximum dose of 1000 mg or, in the United States, as 2 separate infusions of 750 mg administered ≥ 1 week apart [72,73]. In the United States, FDI is also administered up to a maximum dose of 1000 mg within a single infusion; however, in Europe, FDI is dosed according to body weight up to 20 mg/kg [77,78]. The need for fewer infusions with FDI compared with FCM to achieve the target iron dose, coupled with the lack of requirement for serum phosphate monitoring, may lead to overall fewer interactions with the health care system; thus, FDI may represent a more cost-effective option compared with FCM [80,81,82,83], along with an associated quality-of-life benefit [52]. Several cost-utility analyses funded by Pharmacosmos A/S (the manufacturer of FDI) indicate that the requirement for fewer infusions, together with the lower costs associated with monitoring and treating hypophosphatemia, leads to a reduction in direct health care expenditure and that the decrease in hypophosphatemia-related fatigue leads to an increase in quality-adjusted life-years [80,81,82,83].

## 9. Conclusions

Hypophosphatemia represents a clinically important adverse event, associated primarily with repeat dosing of IV FCM in patients with IDA at heightened risk for low serum phosphate. The consequences of chronic severe hypophosphatemia are clinically significant but may be avoided through use of alternative IV supplements in patients considered at risk or where repeated iron infusions are anticipated. Physicians should be aware of the consequences of chronic hypophosphatemia in at-risk patients and the necessary approaches to mitigate its occurrence.

## Figures and Tables

**Table 1:** Summary of study design and primary outcomes of FCM versus FDI clinical studies [44,49] *.

Study	Design and Participants	Treatment	Incidence of Hypophosphatemia		Secondary Endpoints
PHOSPHARE IDA04 and IDA05 [44]	Two open-label, randomized clinical trials conducted in the United States in 245 adults with IDA (Hb ≤ 11 g/dL and serum ferritin ≤ 100 ng/mL) and history of intolerance or unresponsiveness to oral iron	FDI (1000 mg single dose on day 0) FCM (750 mg on day 0 and day 7)	PHOSPHARE IDA04 FDI: 7.9% FCM: 75.0%	Adjusted rate difference: −67.0% (95% CI, −77.4%, −51.5%); *p* < 0.001	Intact FGF23 Significantly higher with FCM vs. FDI at all postbaseline visits Serum phosphate FCM induced reductions of significantly larger magnitude than FDI Urinary phosphate excretion Significantly higher with FCM vs. FDI 1,25-dihydroxyvitamin D Significantly greater decreases with FCM compared with FDI Parathyroid hormone Significantly higher with FCM vs. FDI Iron and anemia parameters Both treatments similarly increased Hb, Hb per gram of iron infused, ferritin, and TSAT
			PHOSPHARE IDA05 FDI: 8.1% FCM: 73.7%	Adjusted rate difference: −65.8% (95% CI, −76.6%, −49.8%); *p* < 0.001	
PHOSPHARE-IBD [49]	Randomized, double-blind clinical trial conducted at outpatient hospital clinics in Europe in 97 patients with IDA due to IBD (Hb < 13 g/dL and serum ferritin ≤ 100 ng/mL) and history of intolerance or unresponsiveness to oral iron	All patients received a single infusion of FDI or FCM (1000 mg) at baseline and a second dose (500 mg or 1000 mg according to total calculated iron dose) at day 35 Baseline Hb < 10 g/dL and bodyweight < 70 kg: total iron dose = 1500 mg Baseline Hb < 10 g/dL and bodyweight ≥ 70 kg: total iron dose = 2000 mg Baseline Hb ≥ 10 g/dL: total iron dose = 1500 mg	Days 0–35 FDI: 8.3% FCM: 51.0%	Adjusted risk difference: −42.8% (95% CI, −57.1%, −24.6%); *p* < 0.0001	Intact FGF23 Intact FGF23 concentrations significantly increased after both FCM infusions compared with FDI Serum phosphate Decreases were significantly greater with FCM vs. FDI Urinary phosphate excretion Higher with FCM vs. FDI 1,25-dihydroxyvitamin D Significantly greater decreases with FCM compared with FDI Parathyroid hormone Significantly greater change from baseline with FCM compared with FDI Iron and anemia parameters Both treatments similarly increased Hb, ferritin, and TSAT
			Days 0–70 (secondary endpoint) FDI: 12.5% FCM: 59.2%	Adjusted risk difference: −46.6% (95% CI, −60.9%, −28.1%); *p* < 0.0001	

FCM, ferric carboxymaltose; FDI, ferric derisomaltose; IDA, iron-deficiency anemia; Hb, hemoglobin; CI, confidence interval; FGF23, fibroblast growth factor 23; TSAT, transferrin saturation; IBD, inflammatory bowel disease. * All study endpoints were biochemical changes in serum variables. Assessment of the clinical consequences of change in serum phosphate levels was precluded due to the short follow-up duration (35–70 days).

**Table 2:** Dosing recommendations for FCM and FDI in the United States and Europe [72,73,77,78].

	United States	Europe
FCM	Approved in adults and children ≥ 1 year of age Body weight ≥ 50 kg 2 × 750 mg dose separated by ≥7 days, or A single dose of 15 mg/kg up to a maximum dose of 1000 mg Body weight < 50 kg Total iron dose of 15 mg/kg administered as 2 infusions ≥ 7 days apart	Approved in adults and children ≥ 1 year of age Adults and adolescents > 14 years of age Maximum single dose is 20 mg/kg not exceeding a total dose of 1000 mg; if total iron need is higher, a second dose can be administered ≥ 7 days apart from initial dose Children 1–13 years of age Single dose of 15 mg/kg not exceeding a total iron dose of 750 mg; if total iron need is higher, a second dose can be administered ≥ 7 days apart from initial dose
FDI	Approved in adults Body weight ≥ 50 kg Single 1000 mg dose Body weight < 50 kg Single infusion of 20 mg/kg iron Repeat dosing if IDA reoccurs	Approved in adults Administered as a single infusion up to 20 mg/kg or as weekly infusions until cumulative target iron dose is achieved If total iron need exceeds that achieved using a 20 mg/kg infusion, the dose must be split into 2 administrations ≥ 7 days apart

FCM, ferric carboxymaltose; FDI, ferric derisomaltose; IDA, iron-deficiency anemia.

## Data Availability

No new data were created or analyzed in this study. Data sharing is not applicable to this article.

## Abbreviations

The following abbreviations are used in this manuscript:

IV	Intravenous
IDA	Iron-deficiency anemia
FCM	Ferric carboxymaltose
Hb	Hemoglobin
ICU	Intensive care unit
FDI	Ferric derisomaltose
CARPA	Complement activation-related pseudoallergy
IgE	Immunoglobulin E
FGF23	Fibroblast growth factor 23
1,25(OH)_2_D	1,25-dihydroxyvitamin D
NaPi	Sodium-dependent phosphate cotransporter
PTH	Parathyroid hormone
CI	Confidence interval
TSAT	Transferrin saturation
IBD	Inflammatory bowel disease
